# Supplementary material for: What does it take to learn the rules of RNA base pairing? A lot less than you may think
Source: bioRxiv. 2025 Aug 2:2025.07.31.668042. Preprint. [Version 1] doi: 10.1101/2025.07.31.668042 (PMC12324431; doi:10.1101/2025.07.31.668042)
Supplement: Supplement 1 [file media-1.gz › supplemental_material/src/rscape_v2.6.0.tar.gz!/rscape_v2.6.0/lib/infernal/Manuscripts/inf-1-appnote/Proofs/licence.pdf]

Licence to Publish

OXFORD JOURNALS
OXFORD UNIVERSITY PRESS

Dear Author

We need your written permission before we can publish your accepted article in *Bioinformatics*.

Please read this form carefully and if you are happy with its terms complete it and immediately fax a copy (both pages) to +44 (0)1865 355741 then send the ORIGINAL to us at the following address**:**

***Bioinformatics*. Oxford Journals. Oxford Universitv Press. Great Clarendon Street. Oxford OX2 6DP. UK.**

You are required to TICK ONE BOX IN STEP 1 TICK ONE BOX IN STEP 2 AND SIGN UNDER STEP 3. In some cases, as explained in STEP3, you need to arrange for your employer to sign as well.

DETAILS (please complete)

Journal: *Bioinformatics* ______________________ Manuscript number: ______________

Article title ______________________

Author(s) ______________________

STEP 1 – THE LICENCE TO PUBLISH

Please tick either Box A or Box B

☐ BOX A: to be completed if copyright belongs to the Author or the Author's employer.

You hereby grant to OUP an exclusive licence of the full period of copyright throughout the world. In particular, this provides OUP with the exclusive right:

- to publish the final version of the Article in the above Journal, and to distribute it and/or to communicate it to the public, either within the Journal, on its own, or with other related material throughout the world, in printed, electronic or any other format or medium whether now known or hereafter devised;
- to make translations and abstracts of the Article and to distribute them to the public; to authorize or grant licences to third parties to do any of the above;
- to deposit copies of the Article in online archives maintained by OUP or by third parties authorized by OUP.

You authorize us to act on your behalf to defend the copyright in the Article if anyone should infringe it and to register the copyright of the Article in the US and other countries, if necessary.

In the case of a multi authored article, you confirm that you are authorized by your co-authors to enter this licence on their behalf

☐ BOX B: to be completed if the Article is protected by UK Crown Copyright, is in the public domain (e.g. US Government Publications) or cannot be fully licensed as requested above. Please briefly indicate the reasons here.

____

____

____

STEP 2 – *OXFORD OPEN*

Please tick either Box A or Box B

☐ BOX A: Please tick this box if you DO NOT wish to **pay to** have your Article published under the Oxford University Press's Open Access option:

Published under the Oxford University Press's open access model, *Oxford Open*. This means that your article will be published in the Journal with standard subscription-based access and you will not be required to pay an open access charge.

☐ BOX B: Please tick this box if you DO wish to **pay to** have your Article published under the Oxford University Press's Open Access option:

This means that your Article will be made freely available online for non-commercial use immediately upon publication **but there is a charge for this which is payable by you**. If you choose this option we require that you complete an open access charge form found at http://www.oxfordjournals.org/oxfordopen/forms and submit it to us as soon as possible.

By indicating that you DO wish to have your Article published under the Oxford University Press's Open Access option you agree to pay the relevant open access charge on the terms set out on the open access charge form submitted by you. For more information about Oxford Open, including a scale of current charges, please go to http://www.oxfordjournals.org/oxfordopen/.

Once published under the open access model, this article will be distributed under the terms of the Creative Commons Attribution Non-Commercial Licence (http://creativecommons.org/licenses/by-nc/2.0/uk/) which permits unrestricted non-commercial use, distribution, and reproduction in any medium, provided the original work is properly cited.

Licence to Publish – continued

STEP 3 – SIGNATURE *Bioinformatics*

You confirm to OUP that the Article (1) is your original work; (2) has not previously been published (in print or electronic format) is not currently under consideration by another journal or if it has already been submitted to other journals, it will be immediately withdrawn; (3) will not be submitted for publication to any other journal following acceptance in the above Journal; and (4) OUP will be the first publisher of the Article.

You warrant to OUP that (1) no part of the Article is copied from any other work, (2) you have obtained ALL the permissions required (for print and electronic use) for any material you have used from other copyrighted publications in the Article; and (3) you have exercised reasonable care to ensure that the Article is accurate and does not contain anything which is libellous, or obscene, or infringes on anyone's copyright, right of privacy, or other rights.

Signature (on behalf of all co-authors (if any)) ____________________

Print name ____________________

Date ____________________

If your employer claims copyright in your work, this form must also be signed below by a person authorized to sign for and on behalf of your employer as confirmation that your employer accepts the terms of this licence.

Signature (on behalf of the employer of the author(s)) ____________________

Print name of employer ____________________

Date ____________________

FURTHER INFORMATION

(Full details of OUP's publication rights policies, including author rights can be found at http://www.oxfordjournals.org/access_purchase/publication_rights.html)

USE OF PRE-PRINTS AND POST-PRINTS (as defined in the Notes below)

On publication of your Article in the Journal you are not required to remove any previously posted PRE-PRINT versions from your own personal website or that of your employer or free public servers of pre-prints and/or articles in your subject area, provided (1) you include a link (url) to the published version of the Article on the Journal's website; AND (2) the Journal is attributed as the original place of publication with the correct citation details given.

You may post the POST-PRINT version of the Article (but not the published version itself) onto your own website, your institution's website and in institutional or subject-based repositories, such as PubMed Central or the Oxford Eprints service, PROVIDED THAT it is not made publicly available until 12 MONTHS after the online date of publication, and that: (1) you include a link (url) to the published version of the Article on the Journal's website; (2) the Journal is attributed as the original place of publication with the correct citation details given.

If the article is published in OXFORD OPEN, provided you have paid the appropriate open access charge, you may deposit the POST-PRINT and/or the finally published version of the article into an institutional or centrally organized repository, immediately upon publication PROVIDED THAT (1) you include a link (url) to the published version of the Article on the journals' website; and (2) the Journal is attributed as the original place of publication with the correct citation details given.

FREE LINK TO PUBLISHED ARTICLE

On publication of your article, you will receive a URL, giving you access to the published article on the Journal website, and information on use of this link.

EDUCATIONAL USE

You may use the Article within your employer's institution or company for educational or research purposes only, including use in course-packs, as long as: (1) you do not use it for commercial purposes or re-distribution outside of the institution/company; (2) you acknowledge the Journal as the original place of publication with the correct citation details given.

Notes (please read)

1 No article will be published unless a licence form has been signed. As the Author(s), copyright in the Article remains yours (or your employer's if your employer claims copyright in your work).

2 Full details of Oxford Journals' Publications Rights Policies including self-archiving rights for authors can be found at http://www.oxfordjournals.org/access_purchase/publication_rights.html and/or in the Instructions to Authors for the journal concerned.

3 Contact details for Permissions: Rights and New Business Development, Oxford Journals, Oxford University Press, Great Clarendon Street, Oxford OX2 6DP Tel: +44 (0) 1865 353561; Email: journals.permissions@oxfordjournals.org http://www.oxfordjournals.org/access_purchase/rights_permissions.html

4 Pre-Print: an unrefereed manuscript version of the article, as submitted for review by a journal

6 Post-Print: A post-print is the final draft author manuscript, as accepted for publication by a journal, including modifications based on referees' suggestions but before it has undergone copyediting and proof correction.
